# Referral rate of patients with incidental gallbladder cancer and survival: outcomes of a multicentre retrospective study

**DOI:** 10.1093/bjsopen/zrae013

**Published:** 2024-03-21

**Authors:** Mike van Dooren, Elise A J de Savornin Lohman, Rachel S van der Post, Joris I Erdmann, Frederik J H Hoogwater, Bas Groot Koerkamp, Peter B van den Boezem, Philip R de Reuver

**Affiliations:** Department of Surgery, Radboudumc, Nijmegen, The Netherlands; Department of Surgery, Radboudumc, Nijmegen, The Netherlands; Department of Pathology, Radboudumc, Nijmegen, The Netherlands; Department of Surgery, Amsterdam University Medical Centers, Amsterdam, The Netherlands; Department of Hepatobiliary and Transplant Surgery, University Medical Center Groningen, Groningen, The Netherlands; Department of Surgery, Erasmusmc, Rotterdam, The Netherlands; Department of Surgery, Radboudumc, Nijmegen, The Netherlands; Department of Surgery, Radboudumc, Nijmegen, The Netherlands

## Abstract

**Background:**

Treatment outcomes of incidental gallbladder cancer generally stem from tertiary referral centres, while many patients are initially diagnosed and managed in secondary care centres. Referral patterns of patients with incidental gallbladder cancer are poorly reported. This study aimed to evaluate incidental gallbladder cancer treatment in secondary centres, rates of referral to tertiary centres and its impact on survival.

**Methods:**

Medical records of patients with incidental gallbladder cancer diagnosed between 2000 and 2019 in 27 Dutch secondary centres were retrospectively reviewed. Patient characteristics, surgical treatment, tumour characteristics, referral pattern and survival were assessed. Predictors for overall survival were determined using multivariable Cox regression.

**Results:**

In total, 382 patients with incidental gallbladder cancer were included. Of 243 patients eligible for re-resection (pT1b–pT3, M0), 131 (53.9%) were referred to a tertiary centre. The reason not to refer, despite indication for re-resection, was not documented for 52 of 112 non-referred patients (46.4%). In total, 98 patients underwent additional surgery with curative intent (40.3%), 12 of these in the secondary centre. Median overall survival was 33 months (95% c.i. 24 to 42 months) in referred patients *versus* 17 months (95% c.i. 3 to 31 months) in the non-referred group (*P* = 0.019). Referral to a tertiary centre was independently associated with improved survival after correction for age, ASA classification, tumour stage and resection margin (HR 0.60, 95% c.i. 0.38 to 0.97; *P* = 0.037).

**Conclusion:**

Poor incidental gallbladder cancer referral rates were associated with worse survival. Age, performance status, resection margin or tumour stage should not preclude referral of a patient with incidental gallbladder cancer to a tertiary centre.

## Teaser

In this multicentre retrospective cohort study, the survival of 382 incidental gallbladder cancer patients treated in secondary care hospitals is reported. This paper gives surgeons guidance on the optimal patient trajectory for incidental gallbladder cancer patients. Patients’ age, performance status, resection margin or tumour stage should not preclude referral of an incidental gallbladder cancer patient to a tertiary centre.

## Introduction

Gallbladder cancer (GBC) is the most common biliary tract malignancy. The prognosis is poor, with an overall 5-year survival rate of less than 5%^1^. In Western populations, up to 70% of GBC cases are diagnosed incidentally (iGBC), during or after cholecystectomy for a benign indication, such as cholecystolithiasis or cholecystitis^2,3^.

Re-resection of the gallbladder bed and lymphadenectomy is associated with better survival. In a recent cohort of 463 iGBC patients from The Netherlands, residual disease was found in 35% of patients and survival in re-resected patients improved from 14 to 53 months^3^. Current guidelines recommend additional resection of the gallbladder bed and hepatoduodenal lymph nodes in iGBC patients with T1b or higher-stage disease, unless contraindicated due to poor performance status or the presence of metastatic disease^4,5^.

Re-resection is carried out in specialized tertiary care centres^6,7^, but studies show that re-resection rates remain low^8–11^. The above-mentioned Dutch study showed that only 25% of eligible patients underwent a re-resection^3^. Determining the rationale behind non-referral and subsequent potential undertreatment of iGBC patients might improve care for iGBC patients.

The present study aims to analyse the referral pattern and outcome of treatment of iGBC patients and the impact of referral on survival in patients staged T1b–T3, eligible for re-resection.

## Methods

### Patient inclusion and data collection

This is a retrospective, multicentre cohort study. This study was approved by the Netherlands Cancer Registry (NCR) Ethical Review Board and a waiver for ethical approval was provided by the Medical Ethics Review Committee of the region Arnhem-Nijmegen (number 2019–5521). The STROBE statement of observational cohort studies was followed^12^. GBC patients were identified from the NCR, which contains data on all new malignancies in The Netherlands. The NCR is maintained by the Netherlands Comprehensive Cancer Organization (IKNL) and is notified of new cancer diagnosis by the automated pathological archive (PALGA)^13^, the nationwide histo- and cytopathological network of The Netherlands, and supplemented by data from the National Archive of Hospital Discharge Diagnosis. Patients diagnosed with iGBC between 2000 and 2019 were included from 27 participating secondary care centres.

### Data collection and variable definitions

Per centre, a local principal investigator was appointed. The local investigator submitted a request to the NCR to provide patient identification numbers of patients with GBC treated at the respective centre. Subsequently, the local investigator reviewed the medical records of identified GBC patients. Only GBC patients diagnosed before or after surgery by histopathological examination were included. Data on treatment and outcomes after referral to a tertiary centre was drawn from the medical records. iGBC patients were excluded when the primary surgery took place in a tertiary care centre. Demographic and clinical characteristics, ASA Physical Status Classification^14^, surgery details and pathology results, referral status and survival data were collected by the local principal investigator by using a standardized pseudonymized case report form in CastorEDC^15^. In this case report form, data were pseudonymized for the lead investigators. Referral pattern was defined as either referral or non-referral to a tertiary care centre for further treatment. Patient medical records were reviewed to identify reasons for non-referral. Patients were eligible for re-resection when pathological T stage was T1b, T2 or T3 and there were no distant metastases, following the guidelines^16,17^. Regional lymph nodes were defined as lymph node located along the cystic duct, common bile duct, hepatic artery or portal vein, in accordance with the AJCC Cancer Staging Manual^18^. Overall survival (OS) was defined as the time from a landmark time of 3 months after the date of primary surgery to the date of death or last follow-up.

### Outcomes

The primary endpoint was OS, defined as the time from the landmark time to the date of death. To assess the impact of referral on OS, patients were classified into two groups: referred and non-referred patients.

To analyse the effect of the Dutch guidelines in 2013, referral rate for patients eligible for re-resection was calculated separately for patients with primary surgery before 2013 and patients with primary surgery in or after 2013.

### Statistical analysis

Categorical variables were reported as counts with percentages and continuous variables as median values with corresponding interquartile ranges (i.q.r.). Differences in median age between referred and non-referred patients were assessed using the Mann–Whitney *U* test, and the difference in age distribution between the groups was assessed using the Wald–Wolfowitz runs test. Differences in baseline variables were assessed using the chi-squared test. Three- and five-year OS were defined as the percentage of patients that were alive respectively 3 and 5 years after diagnosis. Kaplan–Meier estimates of survival were obtained. OS was compared between patient groups using log-rank testing. Univariable and multivariable analysis using backwards logistical regression were used to identify predictive factors for survival in patients eligible for re-resection. In multivariable analysis, tumour stage at primary surgery, ASA status, age (categorized as below or above 65 years at time of diagnosis) and referral to a tertiary care centre were included as factors.

## Results

### Patient characteristics

A total of 382 patients with iGBC diagnosed in a secondary care centre were included in the study. Median age at diagnosis was 69 years (i.q.r. 61–77 years), and 248 patients (64.9%) were female (*Table 1*).

**Table 1:** Characteristics of patients with incidental gallbladder carcinoma (*n* = 382)

Characteristic	Number of patients (%)
Age at diagnosis (years)*	69 (61–77)
**Sex**	
Male	134
Female	248
**ASA class**	
I/II	261 (68.3)
III/IV	94 (24.6)
Unknown	27 (7.1)
**Primary surgery**	
Indication primary surgery†	
Cholecystolithiasis	266 (69.6)
Cholecystitis	113 (29.6)
Gallbladder wall abnormality‡	36 (9.4)
Other§	17 (4.5)
Primary surgery type	
Laparoscopic cholecystectomy	204 (53.4)
Open cholecystectomy	34 (8.9)
Converted cholecystectomy	81 (21.2)
Other¶	51 (13.4)
Unknown	12 (3.1)
Perioperative suspicion of GBC	
Reported	73 (19.1)
Unreported	297 (77.7)
Unknown (no detailed surgery report)	12 (3.1)
**Pathology**	
Tumour size (mm)* (*n* = 109)	23 (15–30)
T stage	
is/1a	39 (10.2)
1b/2/3	262 (68.6)
4	16 (4.2)
x	65 (17.0)
Morphology	
Adenocarcinoma	310 (81.2)
Other	41 (10.7)
Unknown	31 (8.1)
Resection margin	
R0	166 (43.5)
R1/R2	153 (40.0)
Unknown	63 (16.5)
Regional lymph node metastasis	
N0	29 (7.6)
N1	43 (11.3)
Nx	310 (81.2)
Metastasis	
M0	71 (18.6)
M1	29 (7.6)
Mx	282 (73.8)

*Values are median (i.q.r.). †Multiple indications could be present. ‡Polyp or other irregularity on radiological imaging. §Such as biliary pancreatitis, acute abdominal pain, etc. ¶Such as subtotal cholecystectomy, explorative laparotomy or laparoscopy, etc. GBC, gallbladder cancer.

### Primary surgery

The most common indications for cholecystectomy in patients were cholecystolithiasis without cholecystitis (*n* = 221, 57.9%) and cholecystitis (*n* = 113, 29.6%). Suspicion of gallbladder malignancy during primary surgery was described in 73 patients (19.1%). Most patients underwent laparoscopic cholecystectomy (*n* = 204, 53.4%). Primary open cholecystectomy was performed in 34 (8.9%) and conversion from laparoscopic to an open procedure in 81 patients (21.2%) (*Table 1*).

### Histopathology

Tumour morphology was adenocarcinoma in 310 patients (81.2%). Margin status was reported in 319 patients (83.5%). Of these, radical resection was achieved in 166 (52.0%). Regional lymph node metastases were found in 43 patients (11.3%). Distant lymph node or organ metastases were found in 29 patients (7.6%) (*Table 1*).

### Decision-making

Referral pattern was reported in 370 patients. Of these, 127 (33.8%) were not referred to a tertiary care centre due to pTis, pT1a, cT4, pT4 and/or M1 disease. Of 243 patients with an indication for re-resection (pT1b, pT2 or pT3 stage and no metastases), 131 patients (53.9%) were referred to a tertiary care centre and 112 (46.1%) were not.

**Fig. 1:**
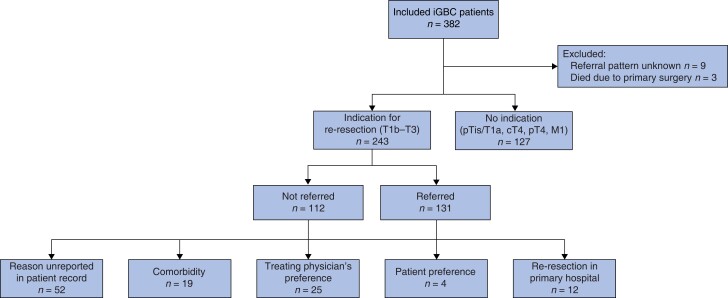
Decision-making for referral to a tertiary care centre in iGBC patients iGBC, incidental gallbladder cancer

Of 112 non-referred patients with an indication for re-resection, 12 patients (10.7%) were not referred because additional surgery was performed at the secondary care centre. In 52 patients (46.4%), the reason for not referring was missing. For 60 patients (53.6%), a reason for not referring was provided: co-morbidities in 19 patients (17.0%), treating physician’s preference in 25 patients (22.3%) and patient preference in four (3.6%) (*Fig. 1*).

Of 165 patients eligible for re-resection with primary surgery before 2013, 68 (41%) were referred to a tertiary care centre, compared with 63 of 82 patients (77%) with primary surgery in or after 2013 (*P* < 0.001).

For patients eligible for re-resection (*n* = 243), baseline characteristics were compared between the referred and non-referred groups (*Table 2*). Referred patients were younger with a median age at diagnosis of 65 years (i.q.r. 57–70) *versus* 74 years (i.q.r. 65–81) in the non-referred group (*P* < 0.001) and more often had ASA class I or II (77.1% *versus* 59.8%, *P* = 0.013). Patients were referred more frequently if they had undergone a non-radical primary resection (48.1% *versus* 25.0%, *P* < 0.001) or had a higher T stage (*P* = 0.006). The primary cholecystectomy was completed laparoscopically in 65.6% (*N* = 86) of the referred group *versus* 44.6% (*N* = 50) of the non-referred group (*P* = 0.001). Cholecystectomy was converted to an open procedure more often in the non-referred group (30.4% *versus* 18.3%) (*P* = 0.028).

**Table 2:** Baseline characteristics of incidental gallbladder carcinoma (iGBC) patients eligible for re-resection (pT1b–pT3, M0) referred or not referred to a tertiary hospital (*n* = 243)

Characteristic	Referred (*n* = 131)¶	Not referred (*n* = 112)¶	*P* value#
Median age at diagnosis (years) (i.q.r.)	65 (57–70)	74 (65–81)	**<0.001**
**Sex**			
Male	42	47	0.110
Female	89	65	
**ASA class**			**0**.**013**
I/II	101 (77.1)	67 (59.8)	
III/IV	23 (17.6)	33 (29.5)	
Unknown	7 (5.3)	12 (10.7)	
**Primary surgery**			
Indication primary surgery*			
Cholecystolithiasis without cholecystitis	95 (72.5)	68 (60.7)	0.051
Cholecystitis	37 (28.2)	40 (35.7)	0.212
Gallbladder wall abnormality†	12 (9.2)	15 (13.4)	0.295
Other‡	1 (0.8)	3 (2.7)	0.242
Primary surgery type			
Laparoscopic cholecystectomy	86 (65.6)	50 (44.6)	0.001
Open cholecystectomy	11 (8.4)	14 (12.5)	0.294
Converted cholecystectomy	24 (18.3)	34 (30.4)	0.028
Other	8 (6.1)	11 (9.8)	0.282
Unknown	2 (1.5)	3 (2.7)	0.528
Perioperative suspicion of GBC	12 (9.2)	17 (15.2)	0.145
**Pathology**			
T stage			0.006
1b	10 (7.6)	23 (20.5)	
2	69 (52.7)	51 (45.5)	
3	34 (26.0)	19 (17.0)	
Unknown§	18 (13.7)	19 (17.0)	
Morphology			
Adenocarcinoma	117 (89.3)	96 (85.7)	0.395
Other	7 (5.3)	9 (8.0)	0.399
Unknown	7 (5.3)	10 (8.9)	0.275
Resection margin			<0.001
R0	49 (37.4)	63 (56.3)	
R1/R2	63 (48.1)	28 (25.0)	
Unknown	19 (14.5)	21 (18.8)	
Regional lymph node metastasis			0.551
N0	10 (7.6)	10 (8.9)	
N1	12 (9.2)	17 (15.2)	
Nx	109 (83.2)	85 (75.9)	

*Multiple indications could be present. †Polyp or other irregularity on radiological imaging. ‡Such as biliary pancreatitis, acute abdominal pain, etc. §T stage was 1b, 2 or 3 but not further specified or distinction could not be made. iGBC, incidental gallbladder cancer. #*P* values lower than 0.05 were marked as bold text. ¶Values are *n* (%) unless otherwise indicated.

### Re-resection procedures and histopathology

Of 243 patients with an indication for re-resection, details about re-resection were missing for 18 (7.4%). Of the remaining 225 patients, 98 (43.6%) underwent additional surgery. Median time from primary surgery to additional surgery was 66 (48–78) days. Staging laparoscopy was performed in 10 (10.2%). Metastatic disease was found in four of ten patients who underwent staging laparoscopy. Of the 94 patients who underwent explorative laparotomy, unresectable disease was found in 24 patients (25.5%). Re-resection was performed in 70 of 243 patients (28.8%); re-resection surgery type was unknown in three patients (4.3%). A wedge resection of segment IVb and V with lymphadenectomy was performed in 50 patients (71.4%); 1 patient (1.4%) underwent a right hemihepatectomy. The remaining 16 patients (22.9%) underwent other resection types, such as solely a wedge resection or lymphadenectomy. Of the 54 patients where the resection margin was described, a radical resection was performed in 47 (87.0%) patients and R1 in seven patients (13.0%). Lymphadenectomy was performed in 62 patients, with a median of three (2–4) nodes evaluated in histopathology reports. In three patients, no nodes were found in the specimen. Lymph node metastases were found in 17 patients (27%).

### Chemotherapy

In the total cohort, adjuvant chemotherapy was described in 14 patients, of which 12 were referred and two were non-referred patients. Gemcitabine and/or cisplatin were part of the adjuvant regimen in 11 patients. Palliative chemotherapy was described in 17 patients, of which five were referred and 12 were non-referred patients. Gemcitabine and/or cisplatin were most used in adjuvant (11 of 14) as well as palliative (11 of 17) regimens. Two patients received chemotherapy for another indication that was not GBC.

### Recurrence

**Fig. 2:**
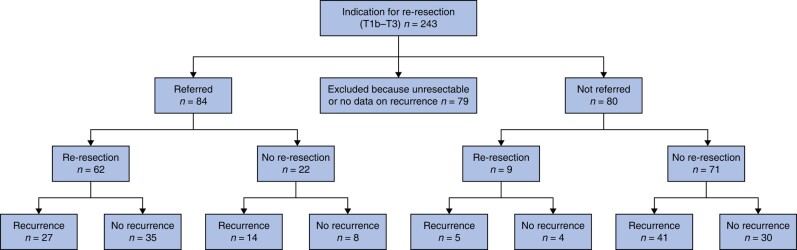
Recurrence in referred and non-referred patients with an indication for re-resection

Of 131 referred patients with an indication for re-resection, 22 (16.8%) were unresectable on additional imaging or during re-resection. Recurrence status at last follow-up was unknown for 25 patients (19.1%). Recurrence was reported in 27 of 62 (43.5%) referred patients that underwent re-resection *versus* 14 of 22 (63.6%) referred patients that did not undergo re-resection (*P* = 0.249). Recurrence was local or in the liver in 27 patients (32.1%), peritoneal in 13 (15.5%) and nodal in 11 patients (13.1%). Recurrence in other organs (for example lung or bones) occurred in 14 patients (16.7%). Recurrence site was not recorded for three patients (3.6%) (*Fig. 2*).

Of 112 non-referred patients with an indication for re-resection, 12 (11%) were unresectable during primary surgery or additional imaging. Recurrence status at last follow-up was unknown for 20 (18%). Recurrence was reported in 41 of 71 (57.7%) of non-referred and not re-resected patients, after a median follow-up of 6.9 (4.6–18.9) months. Recurrence was local or in the liver in 32 patients (45.1%), peritoneal in 11 (15.5%), and nodal in five patients (7.0%). Recurrence in other organs (for example lungs or bones) occurred in 13 patients (18.3%). Recurrence site was not recorded for three patients (4.2%). Of 12 re-resected patients with curative intent in the primary hospital, three showed irresectable disease during re-resection, and recurrence was reported in five of the remaining nine patients after a median follow-up of 5.3 (3.4–17.0) months (*Fig. 2*).

### Survival

Follow-up data was available for 364 patients. Median OS was 21 months (95% c.i. 15 to 27 months). Three-year survival was 35% (*n* = 97) and 5-year survival was 24% (*n* = 60). Of a total of 258 deceased patients, the cause of death could be deduced from medical records in 179 patients (69%). Of those, progression or recurrence of GBC was the cause of death in 152 (85%). Three patients (2%) died due to postoperative complications (two after primary surgery and one after re-resection). Other causes accounted for 24 deaths (13%).

**Fig. 3:**
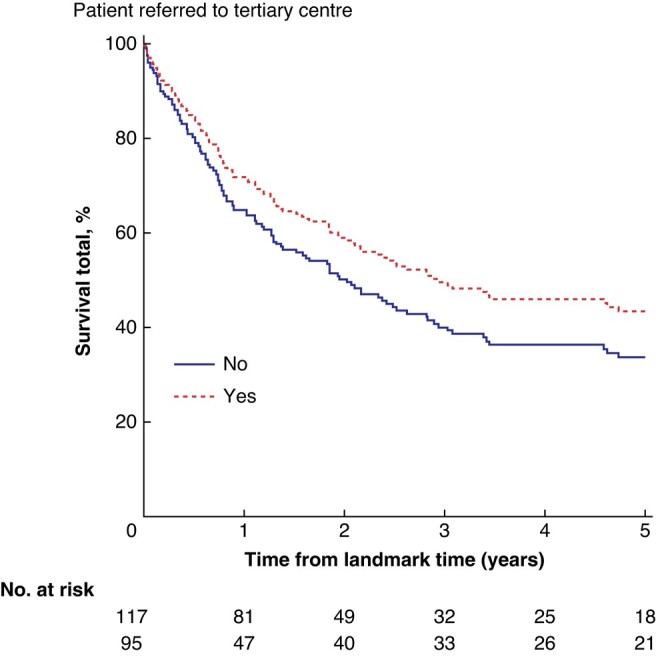
Survival in referred *versus* non-referred group of patients with an indication for surgery (T1b–T3, M0) from a landmark time of 3 months after primary surgery (*n* = 212)

In 247 patients with an indication for re-resection, the median OS was 33 months (95% c.i. 24 to 42 months) in patients referred to a tertiary centre *versus* 17 months (95% c.i. 3 to 31 months) in the non-referred group (*P* = 0.019). Of those patients, 212 underwent follow-up after the landmark time of 3 months. Median OS from the landmark time was 30 months (95% c.i. 17 to 43 months) in referred patients, compared with 25 months (95% c.i. 5 to 45 months) in non-referred patients (*P* = 0.158) (*Fig. 3*).

In multivariable analysis of the patient group with an indication for re-resection, referral to a tertiary care centre was independently associated with superior survival (HR 0.60, 95% c.i. 0.38 to 0.97; *P* = 0.037). ASA classification III/IV (HR 1.80, 95% c.i. 1.14 to 2.84; *P* = 0.012) and a positive resection margin (HR 2.56, 95% c.i. 1.59 to 4.13; *P* < 0.001) were negatively associated with survival. In this model, tumour stage (HR 1.21, 95% c.i. 0.80 to 1.82; *P* = 0.362) and age above 65 years old (HR 1.37, 95% c.i. 0.82 to 2.31; *P* = 0.228) were not independent predictors for survival (*Table 3*). When the 12 patients who underwent additional surgery in the secondary care hospital were included in the non-referred group, referral to a tertiary care centre remained an independent positive factor for survival (HR 0.55, 95% c.i. 0.35 to 0.86; *P* = 0.009).

**Table 3:** Analysis of overall survival in patients with incidental gallbladder carcinoma eligible for re-resection (pT1b–pT3, M0)

Characteristic* (*n*)	Unadjusted HR (95% c.i.)	*P* value†	Adjusted HR (95% c.i.) (*n* = 153)	*P* value‡
Age at diagnosis ≥ 65 years (236)§	1.63 (1.11,2.40)	0.013	1.37 (0.82,2.31)	0.231
ASA III/IV (218)§	1.70 (1.13,2.57)	0.011	**1.80** (**1.14,2.84)**	**0**.**012**
pT stage (200)§	1.34 (0.97,1.86)	0.078	1.21 (0.80,1.82)	0.362
Positive resection margin at primary surgery (197)§	2.19 (1.45,3.30)	<0.001	**2.56** (**1.59,4.13)**	**<0.001**
Referral to tertiary care centre (236)§	0.77 (0.53,1.11)	0.159	**0.60** (**0.38,0.97)**	**0**.**037**

Factors analysed in univariable analysis that were not significant included female sex, indication for primary surgery, open cholecystectomy as primary surgery type, morphology type and regional lymph node metastasis. *Hazard ratios were compared with patients who were negative for the variable for all characteristics, except for pT stage. †*P* value of log-rank testing. ‡*P* value of multivariable Cox regression analysis with backward selection; hazard ratios with *P* value lower than 0.05 are marked as bold text. §This characteristic was added to the multivariable Cox regression analysis. HR, hazard ratio; iGBC, incidental gallbladder cancer.

## Discussion

Referral to a tertiary care centre impacts survival of iGBC patients. Of all patients with an indication for re-resection, only half were referred to a tertiary care centre and a minority (5%) underwent re-resection in their initial hospital. Referral improved significantly after the introduction of national guidelines in 2013. Most frequently, patients were not referred due to the treating physicians’ preference, despite additional surgery being indicated according to treatment guidelines. The present series illustrates that referral benefits survival in iGBC patients.

Non-referral to a tertiary care centre, increased ASA status and a positive resection margin at primary surgery were independently associated with worse survival in iGBC patients.

Treatment guidelines recommend referral to a tertiary care centre for expert evaluation and potential re-resection^3–7,16,17,19^. Recent literature shows, however, that a significant number of patients (>50%) do not undergo re-resection and that referral to a tertiary care centre or to specialized surgical oncologists significantly increases the chances for re-resection^9^. As most larger studies gather data from tertiary care centres, limited data is available on the treatment and referral patterns of patients presenting in secondary care centres. There are no data available on the decision-making process regarding patients who are eligible for re-resection, but not being referred. The present study shows that 29% of iGBC patients with indication for re-resection were referred to a tertiary care centre, which is in line with an earlier study using data from the NCR showing that re-resection was performed in a quarter of iGBC patients with an indication for additional surgery^3^. These outcomes highlight how treating physicians might have a higher threshold for referral in patients expected to benefit less from re-resection, namely frail patients (elderly patients and/or with higher ASA status) or patients with favourable tumour characteristics such as lower T stage and radical primary resection. Co-morbidities and patient preferences accounted for a minority of non-referred patients in the present study. In almost half of the non-referred patients, the reason for non-referral was not clearly outlined. It must be mentioned that Dutch national guidelines have changed during the inclusion interval; no recommendations for iGBC were available before the 2013 version of the guidelines, which might have affected the referral rate^5^.

The present study confirms that referral to a tertiary care centre is an important factor associated with survival benefit for patients eligible for re-resection^3,6,7,20,21^. All patients with iGBC who are eligible for re-resection must be referred to a tertiary care centre for further evaluation. A multidisciplinary team including a surgical oncologist should balance risks and benefits of a potential re-resection in a patient-tailored approach. The establishment of a nationwide expert centre or guideline as well as efforts to increase awareness on treatment of iGBC amongst surgeons might improve referral rates.

Strengths of the present study include its multicentre nature. All data was gathered from secondary care centres and reflects general population characteristics. The main limitation of this study is the retrospective nature and data collection. Treatment patterns, registration and availability of data differ among hospitals, resulting in inhomogeneity and incompleteness of data.

iGBC patients eligible for re-resection have a survival benefit from referral to a tertiary care centre, regardless of their age, ASA class, resection margin or tumour stage. Referral is recommended for all iGBC patients with a potential indication for re-resection. Optimal treatment should always be discussed in a multidisciplinary team meeting.

## Collaborators

Eduard A. van Bodegraven, Evert-Jan Boerma, Andries E. Braat, Rebecca P.M. Brosens, Bart J.G.A. Corten, Tessa C.M. Geraedts, Arja Gerritsen, Frederike A.B. Grimme, Joost T. Heikens, Britt Jansen, Nenke de Jong, Remon Korenblik, Romy S. de Kuijer, Barbara S. Langenhoff, Andreas W.K.S. Marinelli, Jos W.S. Merkus, Lynn E. Nooijen, Ndidi Obihara, Lisanne A.E. Posma, Fay R.K. Sanders, René Scheer, Niels Smakman, Denis Susa, Sophie Taverne, Joost Verhelst, Danielle Verver, Bas S.T. van Vugt, Jeroen L.A. van Vugt, Kevin P. Wevers and Fennie Wit.

## Data Availability

The data sets generated during and/or analysed during the present study are available from the corresponding author on reasonable request.
