# Corrigendum to: Referral rate of patients with incidental gallbladder cancer and survival: outcomes of a multicentre retrospective study

**DOI:** 10.1093/bjsopen/zrae064

**Published:** 2024-05-28

This is a corrigendum to: Mike van Dooren, Elise A J de Savornin Lohman, Rachel S van der Post, Joris I Erdmann, Frederik J H Hoogwater, Bas Groot Koerkamp, Peter B van den Boezem, Philip R de Reuver, Referral rate of patients with incidental gallbladder cancer and survival: outcomes of a multicentre retrospective study, *BJS Open*, Volume 8, Issue 2, April 2024, zrae013, https://doi.org/10.1093/bjsopen/zrae013

In the originally published version of this article, Figure 1 was erroneous.

The article has been updated with the correct version of the figure.

